# Exploring the landscape of digital servitization: A systematic review

**DOI:** 10.12688/f1000research.150946.1

**Published:** 2024-07-18

**Authors:** Hendri Ginting, Hamidah Nayati Utami, Riyadi Riyadi, Benny Hutahayan

**Affiliations:** 1 Brawijaya University, Malang, East Java, Indonesia

**Keywords:** Digital Servitization, Systematic Literature Review, PRISMA, Determinants

## Abstract

**Background:**

Digital servitization is a strategic transformation where companies adopt a service-focused approach in response to the prevailing trend of digitalization. Utilizing digital technology, businesses manage product and service operations and develop new value propositions. Further research into digital servitization is essential for maintaining competitiveness and meeting evolving market demands globally. This study aims to comprehend the evolution of digital services from theoretical and practical viewpoints and examines how these challenges are tackled.

**Methods:**

Utilizing a systematic literature review methodology, the study adopts the PRISMA approach to identify 26 pertinent articles from a pool of 340 papers obtained through a SCOPUS database search. These papers were published between 2019 and 2024 and were retrieved using the keywords (‘digitalization OR digital AND servitization OR servitization’). The selection process involved scrutinizing titles, abstracts, and keywords based on predefined criteria.

**Results:**

The findings reveal that current digital servitization research emphasizes five determinant variables: digitization, servitization, manufacturing servitization, process innovation, and product innovation. Additionally, response variables influenced by digital servitization were identified, including firm competitiveness, firm performance, financial performance, firm profitability, and sustainable performance.

**Discussion:**

The results of this review point to inconsistencies, underscoring the necessity for additional research into the factors influencing digital servitization across companies beyond the manufacturing sector. This highlights the importance of gaining a deeper understanding of digital servitization strategies and their impacts across various industries. By expanding the scope of research to include a broader range of sectors, researchers can provide more comprehensive insights into the complexities and nuances of digital servitization adoption. This broader perspective enables a more thorough examination of the challenges and opportunities associated with implementing digital servitization strategies, ultimately contributing to a richer understanding of its implications for businesses across diverse industries.

## 1. Introduction

In the ever-evolving digital era, companies across various industry sectors face significant pressure to adapt to the rapidly changing business landscape. One of the notable transformations in business strategies is the shift from product-centric models towards digitally-oriented services. Digitalization is compelling many organizations to explore ways of shifting their focus away from a product-centered approach and towards offering services that are digitally oriented ( Kohtamäki et al., 2020; Kowalkowski et al., 2017). By leveraging digital technology, these organizations aim to generate value in their processes and provide improved customer experiences. This effort has led to the emergence of a new concept in servitization literature, known as digital servitization ( Coreynen et al., 2020; Gebauer et al., 2020; Paschou et al., 2020).

Digital servitization involves the use of digital technology to integrate services into existing products, creating added value for customers, and improving operational efficiency. Digital servitization represents a strategic transformation where companies are required to transition from a preplanned, product-centric mindset to a collaborative, service-focused approach in order to adapt to new production methods and collaboration models in the digital age ( Vargo & Lusch, 2017; Chen et al., 2023). In the realm of digitalization, organizations utilize digital technology to efficiently manage their product and service operations, creating innovative value propositions such as intelligent products, services, and solutions ( Vendrell-Herrero et al., 2017). When viewed from the servitization perspective, companies incorporate customer-centric services into their existing product offerings.

Engaging in the digital ecosystem, digital servitization links internal entities and external partners to build a service framework facilitated by technology, ongoing interaction, and procedural collaboration ( Bustinza et al., 2018). In this context, digital products serve as boundary objects within the service system, enabling the integration of resources and activities between service providers and users, which in turn enhances their interaction and collaboration. Through the digital servitization transformation, sophisticated business models are developed through process innovation ( Hilbolling et al., 2022) and collaboration with diverse stakeholders in a more advanced, interactive manner, thereby embedding themselves within the digital ecosystem.

The digital servitization process involves a simultaneous and dynamic interaction between business models and digital technologies ( Chen et al., 2021). This transition is intricate and demands that companies cultivate fresh capabilities and foundational elements at a micro level to facilitate the change ( Chirumalla et al., 2023). The shift towards digital servitization requires organizations to adapt their structures, processes, and cultures to accommodate the new business model ( Sklyar et al., 2019).

Digital servitization results in the creation of digital product service systems, amalgamations of physical products and intangible services tailored to meet the specific requirements of individual customers ( Lerch & Gotsch, 2015). Yet, the body of literature concerning digital servitization remains constrained, primarily concentrating on select digital technologies and particular industries ( Paschou et al., 2020). Hence, additional research is warranted to delve into the potential advantages and areas of application for digital servitization.

However, despite the significant potential associated with digital servitization, numerous challenges must be addressed, such as technology integration, complex data management, information security, and cultural changes within organizations. Therefore, a deeper understanding of this concept through systematic and comprehensive research is required.

In this context, systematic literature research on digital servitization can be a highly valuable tool. It can help identify trends, developments, and weaknesses in the existing literature, as well as provide a clear view of the extent to which this concept has been studied and applied across various industry sectors. Based on this, the Research Questions (RQ) to be answered in this study are as follows:

RQ1: What are the definitions of digital servitization based on previous literature?

RQ2: In which industries is digital servitization extensively researched?

RQ3: What are the determinants variable of digital servitization?

RQ4: What are the factors influenced by digital servitization?

This paper conducts a systematic literature review (SLR) on digital servitization, as SLRs offer a comprehensive summary of prior research on the topic and help identify knowledge gaps, thereby highlighting areas for future investigation. The primary objective of this paper is to enrich the current body of literature on digital servitization in two key ways: first, by presenting a thematically organized classification of existing research, and second, by using the SLR findings to propose detailed factors that influence digital servitization for future research.

## 2. Literature review

The resource-based view (RBV) assumes that a company is an entity that maximizes profits guided by rational managers operating in a specific market, reaching a level that can be predicted and moving towards equilibrium ( Bromiley & Papenhausen, 2003; Leiblein, 2003). RBV emerged to comprehend how combinations of valuable, rare, inimitable, non-substitutable, and organized resources (VRIN/O) can yield competitive advantages for a company ( Barney, 1991; Penrose, 1959). Resources need to be rearranged to capitalize on new business opportunities, such as digital servitization ( Kohtamäki et al., 2019).

Resources are tangible and intangible assets of an organization, where the organization can accumulate resources from external sources that are treated as crucial assets in the production process ( Kraja, 2018). The RBV theory suggests that organizations should focus on managing resources that can generate higher value and disregard some resources with lower opportunities to enhance value propositions ( Wernerfelt, 2014). Huikkola and Kohtamäki (2017) pinpointed essential resources and strategic procedures that establish strategic competencies and competitive edges. These competencies encompass fleet administration, technological advancement, mergers and acquisitions, value evaluation, project oversight, supplier network administration, and cooperative value generation.

The digital aspect of digital servitization presents opportunities for enhancing process and capability development, resulting in enhanced value creation and capture, improved efficiency in customization, and more streamlined order fulfillment processes. It also facilitates effective resource restructuring as companies venture into new business prospects, including untapped customer markets, innovative projects, and smart solutions ( Kohtamäki et al., 2019). Overall, a company’s readiness to adapt to digital servitization will hinge on its history and its capability to restructure both its internal operations and external resource pool ( Sklyar et al., 2019).

## 3. Methods

This article seeks to offer profound insights into digital servitization, identifying emergent key concepts, and delineating the challenges and opportunities encountered by firms considering this approach. Through a systematic literature review, a more comprehensive comprehension of how digital servitization has reshaped corporate operations and customer interactions in the constantly evolving digital age can be attained.

### 3.1 Search strategy

The Preferred Reporting Items for Systematic Reviews and Meta-Analyses (PRISMA) approach was used in this systematic literature review ( Farisyi et al., 2022; Liberati et al., 2009; Moher et al., 2015). The PRISMA approach ensures transparency and clarity in reporting systematic literature reviews by using an evidence-based checklist ( Ginting, 2024b) connected to a four-phase flowchart. By applying the PRISMA approach, one may reduce bias, lessen the impact of chance, and improve the quality of data analysis.

The information search was carried out on a comprehensive academic research database, specifically Elsevier (SCOPUS), which houses extensive collections of scholarly literature. The search included articles published between 2019 and 2024 to ensure the literature used in the research analysis remains current and up-to-date. The search was completed on January 15, 2024.

### 3.2 Inclusion and exclusion criteria

Two sets of keywords associated with the concepts of servitization and digitalization were chosen and utilized in tandem. The selected keywords for this research are as follows: *‘digitalization OR digital AND servitization OR servitisation’.* The Inclusion Criteria (IC) employed as guidelines in conducting the Systematic Literature Review are outlined below:

IC1: Articles are original documents that have undergone a peer-review process and were published between 2019-2024.

IC2: Articles are original documents written in the English language.

IC3: Research that employs quantitative methods.

IC4: Studies aimed at identifying factors influenced by digital servitization.

In detailing the Inclusion Criteria (IC), IC1 emphasizes that only articles that are original documents and peer-reviewed, published between 2019-2024, will be considered. Furthermore, IC2 indicates that the selected articles must be in the English language. This is because research conducted in English generally provides the most current information for that period, ensuring more accurate updates. IC3 ensures that only research using a quantitative approach will be taken into account in this study. Quantitative methods are used to collect numerically measurable data, providing a more structured framework for analyzing relationships between variables and identifying indicators in measuring digital servitization. Meanwhile, IC4 underscores that the studies focused on in this research must have a specific aim of identifying factors influenced by digital servitization. Furthermore, articles that were not fully accessible were excluded from the study.

### 3.3 Data collection

The subsequent step involved identifying publications and conducting practical screening, resulting in the identification of 340 papers. Following screening with IC1 and IC2, 310 studies were excluded, and the remaining studies were moved to the screening phase. At this stage, the titles, abstracts, and keywords of the 310 collected papers were scrutinized for relevance to the research objectives, resulting in 62 studies. Subsequently, the full text of the remaining 62 publications was meticulously reviewed, with only articles deemed capable of contributing to answering the research questions being selected. Ultimately, based on the predefined criteria, namely IC3 and IC4, 26 papers were chosen and analyzed to compile knowledge in this research field and identify potential knowledge gaps and future research directions.

### 3.4 Data items

**Figure 1.**
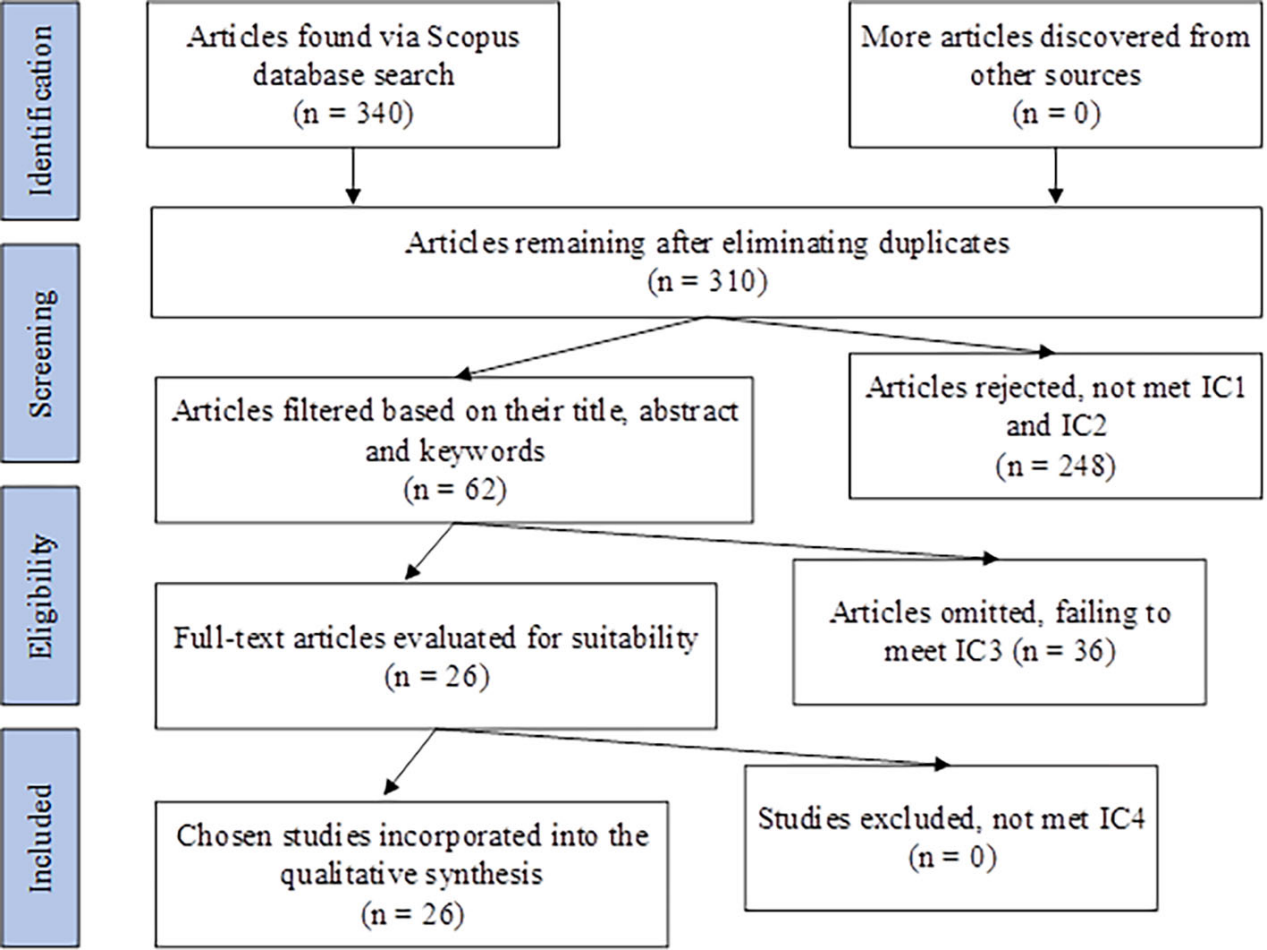
Flowchart illustrating the process of literature selection reviewed using PRISMA.

The information gleaned from each article is outlined systematically, encompassing details such as the publication year, authors, country of origin and sample size, research aims, variables examined, aspects affected by digital servitization, and findings concerning its influence on dependent variables. Figure 1 provides a comprehensive depiction of the stages involved in this systematic literature review ( Figure 1 also uploaded in Ginting (2024a)).

Each study was independently assessed by four authors. To ensure objectivity and reduce the likelihood of bias in the evaluation process, the authors worked independently. Any discrepancies between the authors’ assessments were resolved through discussion and consensus.

### 3.5 Data extraction and synthesis

To determine the degree of information completeness in accomplishing the research objectives, data was gathered from all of the chosen papers. A more thorough summary of the studies that need to be studied and how they can particularly answer the research questions is also provided by the extraction process. These actions are predicated on the previously mentioned selection procedure. After extraction, the data is sorted and entered into a worksheet. Cruzes & Dyba (2011) suggest using thematic synthesis to synthesize the findings. Additionally, an integrated synthesis process technique is used to guarantee that all study goals are met.

## 4. Results

### 4.1 Research results and qualitative synthesis

The SCOPUS database search using the keywords (‘digitalization OR digital AND servitization OR servitization’) produced 340 papers published between 2019 and 2024 in English. These papers underwent examination and selection based on IC2 and IC3 criteria, focusing on titles, abstracts, and keywords, resulting in 62 articles remaining. One article remained inaccessible or uncategorized among those excluded or not researched. Ultimately, 26 articles were left for further analysis after this procedure. Selected articles are uploaded in Ginting (2024c) using Figshare.

**Figure 2.**
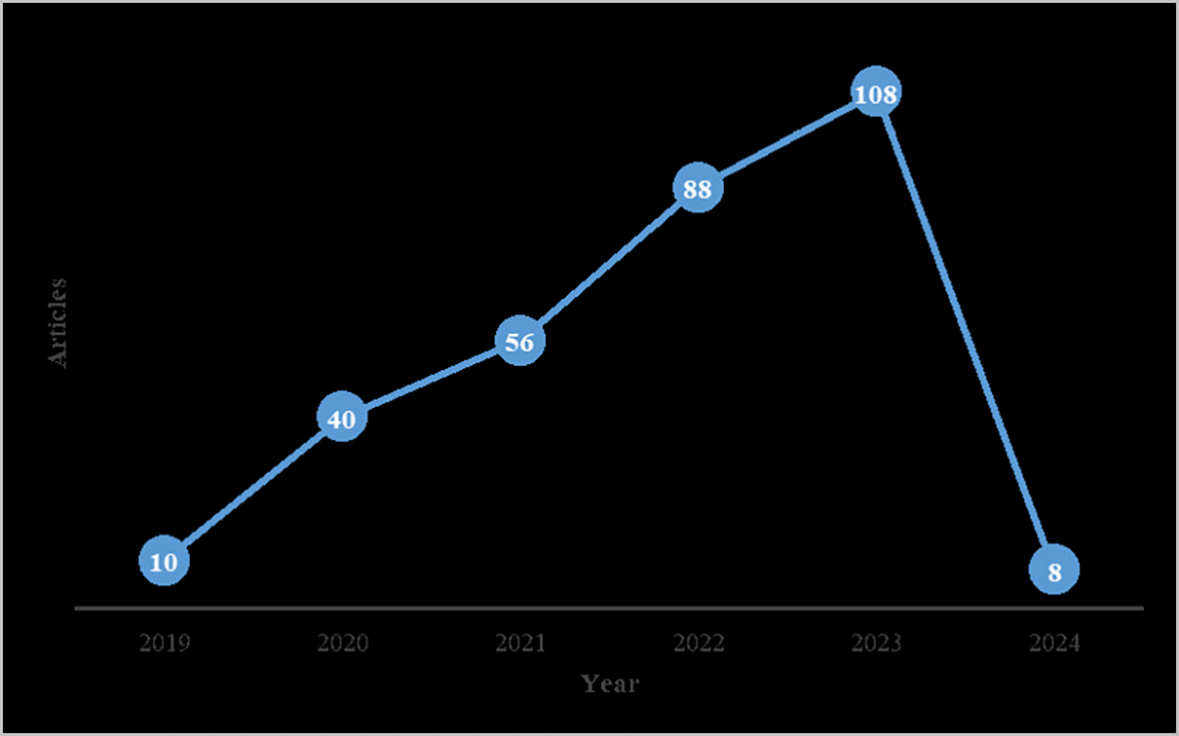
Distribution of selected studies over 5 years.

Numerous journal publications on digital servitization are issued each year, with a notable increase observed in 2019. These journals utilize a combination of qualitative and quantitative methodologies. This trend underscores the ongoing relevance of research on digital servitization in recent years, as illustrated in Figure 2 below.

**Figure 3.**
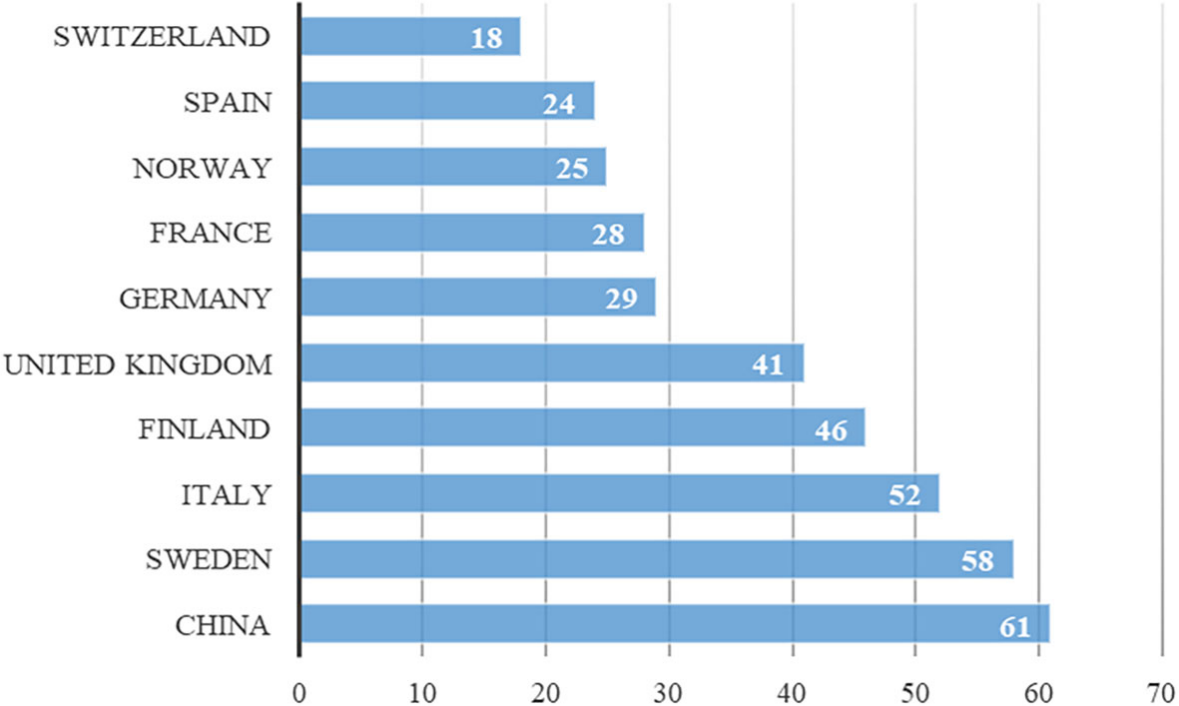
Country production over time.

Between 2019 and 2024, numerous countries, including China, Finland, France, Germany, Italy, Norway, Spain, Sweden, Switzerland, and the United Kingdom, contribute to journals addressing digital servitization. Recent data indicate the ongoing relevance of research on digital servitization in these countries, as illustrated in Figure 3.

**Figure 4.**
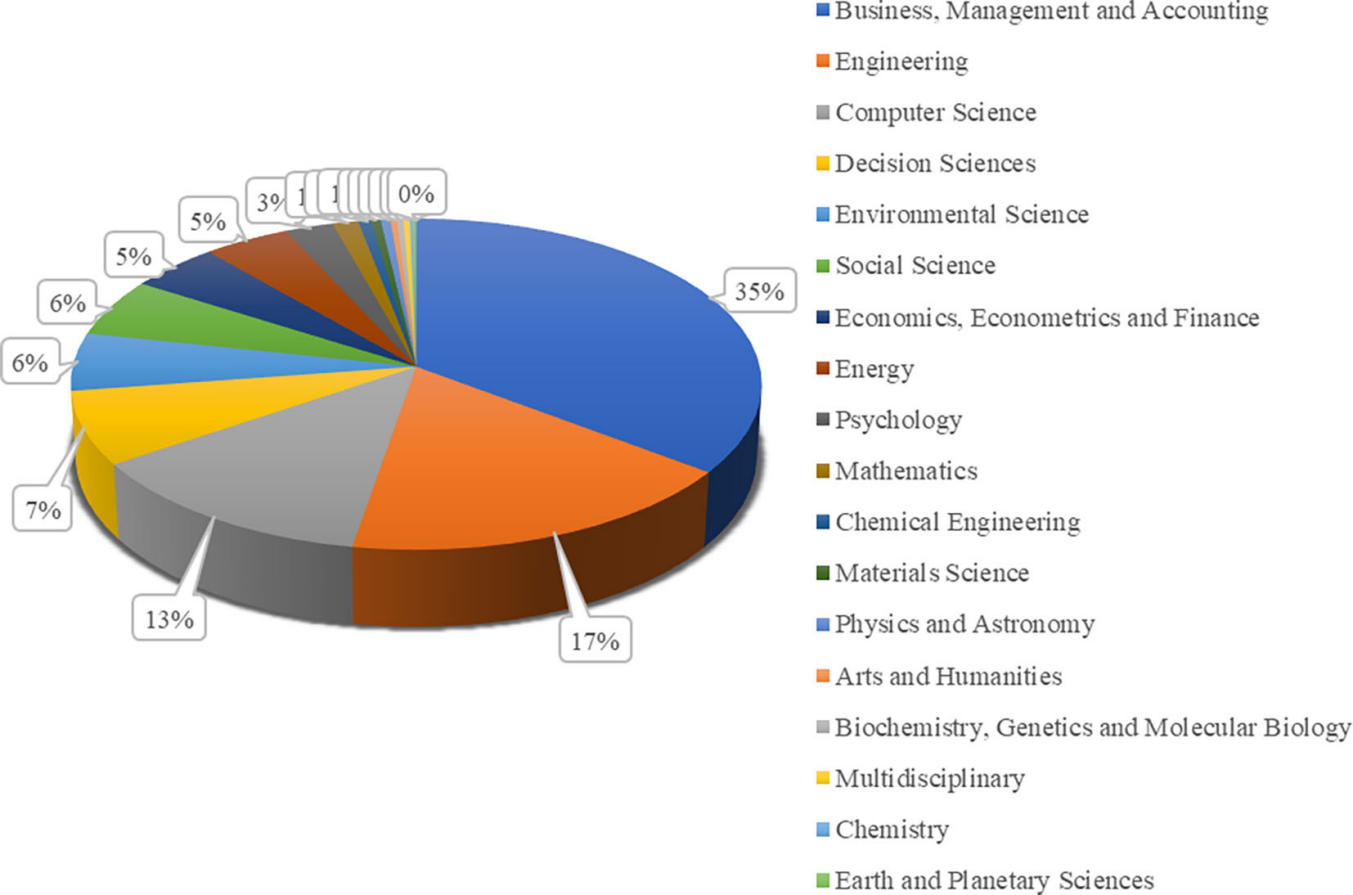
The percentage of scholarly publications based on fields of study.

Based on an analysis of reputable international journals, the percentage data of articles published in 18 major fields of study over the last five years is presented in Figure 4. 35% of the articles focus on business, management, and accounting. Engineering and computer science represent 17% and 13% of the total scholarly publications. The dominance of publications in applied fields such as business, engineering, and computer science reflects the dynamic needs of the industrial world for innovations in business models, technology, and digital solutions in the current era of the Industrial Revolution. Meanwhile, the smaller proportion of articles in decision sciences and other fields indicates the limited scope of multidisciplinary studies and fundamental research in addressing the complex challenges faced by modern society.

Additionally, a qualitative synthesis was conducted on the 26 chosen articles, as illustrated in Table 1.

**Table 1.** List of chosen articles.

No	Authors	Sectors	Country
1	Huang et al. (2024)	Manufacturing industry	China
2	Chen et al. (2023)		
3	Zhou et al. (2023)		
4	Gao et al. (2023)		
5	Miao et al. (2023)		
6	Jiang et al. (2023)		
7	Wang et al. (2023)		
8	Wen et al. (2022)		
9	Chen & Zhang (2021)		
10	Zhao et al. (2022)	Industrial firms	
11	Upadhayay et al. (2024)	Motor Vehicle Equipment, Pharmaceuticals and Medicine and Computers and Electronics industries	Many Countries
12	Kumar et al. (2024)	Firms working in the food and beverage, automotive, and electronic appliance industries that use product-service-based business processes	
13	Behl et al. (2024)	Retail industry	
14	Vendrell-Herrero et al. (2023)	Manufacturing industry	Spanish
15	Opazo-Basáez et al. (2022)		
16	Martín-Peña et al. (2020)	Industrial firms	
17	Rakic et al. (2022)	Manufacturing industry	Serbia
18	Lalic et al. (2020)		
19	Kohtamäki et al. (2024)	Manufacturing industry	US
20	Abou-Foul et al. (2021)	Manufacturing industry	US and European
21	Bortoluzzi et al. (2022)	Manufacturing industry	Italy
22	Mishra et al. (2022)	Manufacturing industry	India
23	Agarwal et al. (2022)	Manufacturing industry	Nordic
24	Coreynen et al. (2020)	Manufacturing industry	Belgia
25	Kohtamäki et al. (2020)	Manufacturing industry	Swedish
26	Pham and Vu (2022)	Public service sector	Vietnam

### 4.2 Systematization for searching dual role of digital servitization

Building upon the analysis of the 26 selected articles, determinants of digital servitization are further scrutinized utilizing the following additional criteria:

1. Digital servitization is used as an independent and dependent variable.
2. Digital servitization is assessed or quantified in diverse forms, serving as both an independent and dependent variable.
3. Determinants and response included as research outcomes are those examined in at least two articles and used as dependent and response variables.

Considering the criteria above, Table 2 presents the determinants, items, result, conclusion, and references related to digital servitization.

**Table 2.** Determinants and response variable of digital sertivization.

No	Determining factor	Item	Result	Conclusion	Prior studies
1	Digitalization	Operational services	Positive	Positive	Abou-Foul et al. (2021), Kohtamäki et al. (2020)
		R&D services	Positive		
		Consulting services	Positive		
2	Servitization	Integration of services into product offerings	Negative	Inconsistent results	Rakic et al. (2022), Bortoluzzi et al. (2022)
		Transition to service-centric	Positive		
		Self-assessment sertivization transition	Positive		
3	Manufacturing Servitization	Proportion of service revenue to total revenue	Positive	Positive	Agarwal et al. (2022), Chen & Zhang (2021)
4	Process Innovation	Number of process innovations introduced	Positive	Positive	Opazo-Basáez et al. (2022), Martín-Peña et al. (2020)
5	Product Innovation	Number of product innovations introduced	Positive	Positive	Opazo-Basáez et al. (2022), Vendrell-Herrero et al. (2023)

Apart from examining digital servitization as a dependent variable, this research also considers it as an independent variable. Table 3 below presents the variable responses, items, results, conclusions, and references related to digital servitization as an independent variable.

**Table 3.** Response variable of digital sertivization.

No	Response factor	Item	Result	Conclusion	Prior studies
1	Firm Competitiveness	Product/service quality	Positive	Positive	Huang et al. (2024), Chen et al. (2023)
		Client satisfaction			
		Good public image			
2	Firm Performance	Sales growth	Positive	Inconsistent results	Zhou et al. (2023), Miao et al. (2023), Rakic et al. (2022), Bortoluzzi et al. (2022), Martín-Peña et al. (2020)
		Profitability			
		Market share			
		Customer satisfaction			
		Return on assets (ROA)	Positive		
		Return on equity (ROE)			
		Tobin’s Q			
		Value added ratio (EVAR)			
		Earnings before interest and taxes (EBIT)			
		Share of revenue from service	Positive Negative		
		Self-assessment effectiveness	Positive		
		Total sales	Positive		
3	Financial Performance	Company’s financial performance	Positive	Positive	Abou-Foul et al. (2021), Kohtamäki et al. (2020)
		Return on assets (ROA)	Positive		
4	Firm Profitability	Earnings before interest and taxes (EBIT)	Positive	Positive	Kohtamäki et al. (2024), Vendrell-Herrero et al. (2023)
		Profit margins	Positive		
5	Sustainable Performance	Community performance	Positive	Positive	Behl et al. (2024), Pham and Vu (2022)
		Economic performance			
		Environmental performance			
		Governance performance			
		Human performance			
		Convenience and efficiency	Positive		
		Customer support			
		Navigation			
		Privacy and security			

## 5. Discussion

### 5.1 RQ1: What are the definitions of digital servitization based on previous literature?

Digital servitization is a multifaceted and continuous procedure, given its amalgamation of servitization and digitalization ( Chen et al., 2021). As businesses increasingly integrate digital technologies and reshape their business models, they must confront the challenges and opportunities inherent in this transition, including adjusting to more dynamic and interconnected markets ( Chen et al., 2021). Digital servitization can occur within ecosystems, where companies work together to create value-creating and capturing systems ( Kohtamäki et al., 2019).

Employing digital technologies like big data and cloud computing empowers companies to gather and analyze extensive datasets, offering valuable insights and stimulating innovation ( Hacioglu, 2019). Digital servitization frequently entails adopting novel business models centered around offering packages comprising products, services, and digital tools like IoT, big data, and cloud computing ( Hacioglu, 2019). Digital servitization enables connectivity between products, services, and customers, allowing for better monitoring, control, and optimization ( Chen et al., 2021).

Digital servitization, as defined by Parate et al. (2022), encompasses leveraging digital technology to elevate existing business models or devise new ones. In order to further develop this idea, Paschou et al. (2020) present a digital servitization maturity model that includes elements like strategy, business procedures, customer experience, and organizational culture. In their contribution to this conversation, Tronvoll et al. (2020) identify three critical turning points that are essential for successful digital servitization: moving from hierarchy to partnership, from scarcity to abundance, and from planning to discovery. Finally, Lerch & Gotsch (2015) examines the fusion of digitalization with service innovation, underscoring the potential ramifications for service provision.

### 5.2 RQ2: In which industries is digital servitization extensively researched?

Research Question 2 (RQ2) delves into the industries where digital servitization has been extensively researched. A review of the literature indicates that, in the context of digital servitization, a wide range of industries have garnered substantial scholarly attention. Digital servitization study finds a dominant domain in the manufacturing industry. Several research papers examine the incorporation of digital technology into conventional manufacturing processes, delving into the ways in which this transition to service-oriented business models impacts the performance, sustainability, and innovation of firms. Another notable industry is the information technology (IT) sector, given its intrinsic connection to digital technologies. Research in this domain often focuses on how IT firms leverage digitalization to offer innovative service solutions, enhance customer experiences, and drive competitive advantage. Additionally, the automotive industry garners considerable attention, with studies exploring how automotive companies embrace digital technologies to offer value-added services alongside traditional product offerings. The healthcare sector is also a significant area of research interest, particularly regarding the adoption of telemedicine, remote patient monitoring, and personalized healthcare solutions. Moreover, the financial services, retail, energy, and telecommunications industries all feature prominently in digital servitization research, reflecting the widespread adoption and impact of digital technologies on business models, value propositions, and customer experiences across diverse sectors. Understanding the nuances of digital servitization within specific industries is crucial for practitioners and policymakers navigating the evolving landscape of service-driven economies.

### 5.3 RQ3: What are the determinants variable of digital servitization?

Research Question 3 (RQ3) aims to discern the determinants variables of digital servitization as elucidated in Table 2. Each determinant variable of digital servitization, in a more complex and comprehensive manner, can be elucidated within the following subsections:

**5.3.1 Digitalization**

Digitalization plays a pivotal role in driving the adoption of digital servitization, as digitally mature companies possess the capabilities and infrastructure essential for implementing such business models. In other words, companies actively employing digital technology are more prepared and inclined to adopt digital servitization strategies ( Vendrell-Herrero et al., 2017). A study conducted by Kohtamäki et al. (2020) on the relationship between digitization and servitization concluded that the level of adoption of digital technology by a manufacturing company significantly affects its ability to implement the digital servitization business model. This is consistent with earlier findings indicating the positive role of operational digitization and digital infrastructure in enabling servitization in the manufacturing industry ( Kohtamäki et al., 2020).

**5.3.2 Servitization**

Servitization pertains to the extent to which services are integrated into the product offerings of a manufacturing company. Coreynen et al. (2020) define servitization as the transformation of a business model from a product-focused approach to a solution-focused one by integrating product-service bundles. The higher the adoption of a hybrid product-service business model, the greater the likelihood of the company implementing digital servitization strategies. Both Bortoluzzi et al. (2022) and Rakic et al. (2022) identified a significant positive correlation between the extent of servitization in manufacturing firms and the adoption of digital servitization. This aligns with a longitudinal study by Coreynen et al. (2020), which concluded that as a company’s age increases, it is typically accompanied by an increase in the complexity of product-service offerings, thereby driving the adoption of digital solutions to integrate and manage these offerings.

**5.3.3 Manufacturing servitization**

Manufacturing servitization, refers to the degree of integrating services into the manufacturing operations and product offerings of an industrial company. Neely (2008) defines *manufacturing servitization* as a phenomenon where manufacturing companies offer additional services related to their products. As the proportion of a company’s revenue derived from services increases, so does the level of manufacturing servitization, providing an opportunity to implement digital servitization to facilitate the delivery of these service values ( Neely, 2008; Visnjic et al., 2018). Agarwal et al. (2022) and Chen and Zhang (2021) both concluded that companies embracing a high degree of manufacturing servitization are also inclined to adopt digital platforms and solutions. The aim is to integrate and present product-service offerings effectively. Put simply, there exists a correlation between a high degree of manufacturing servitization and the adoption of digital technology to streamline and improve efficiency in delivering products and services. Consistent with a study conducted by Visnjic et al. (2018), it is concluded that manufacturing servitization creates new opportunities for implementing digital technology to support the operationalization of business models.

**5.3.4 Process innovation**

Process innovation, refers to the development and introduction of new production, distribution, or management methods by manufacturing companies that significantly enhance the quality and efficiency of their internal processes ( Garcia & Calantone, 2002). The more successful process innovations implemented, the higher the flexibility and operational capabilities of the company to adopt the digital servitization business model to enhance service quality for customers ( Raddats et al., 2014; Valtakoski, 2017). Opazo-Basáez et al. (2022) and Vendrell-Herrero et al. (2023) found that a higher level of process innovation positively influences the ability of manufacturing companies to implement digital servitization strategies. The transformation and improvement of internal production processes through automation serve as a crucial foundation for adopting the digital servitization business model to enhance the value proposition to consumers ( Martín-Peña et al., 2018).

**5.3.5 Product innovation**

Product innovation entails a company’s creation and introduction of new products or services that represent a significant improvement over previous offerings, based on its performance ( Schumpeter & Swedberg, 2021). The more innovative products are introduced, the greater the incentive for manufacturing companies to implement digital servitization strategies to support the delivery of value from these innovative products to consumers ( Frank et al., 2019). Opazo-Basáez et al. (2022) and Vendrell-Herrero et al. (2023) found a positive impact of the number of product innovations on the ability of companies to implement the digital servitization business model. The success of developing smart/connected products requires supporting digital service offerings to integrate and optimize the value of the products for consumers ( Rymaszewska et al., 2017; Kohtamäki et al., 2020).

### 5.4 RQ4: What are the factors influenced by digital servitization?

Research Question 4 (RQ4) aims to understand the variables that can be influenced by digital servitization as described in Table 3. Each variable that can be influenced by digital servitization is discussed in a more complex and comprehensive manner in the following subsections:

**5.4.1 Firm competitiveness**

Developing a unique value proposition that differentiates a business from its rivals is one way to become competitive ( Porter, 1997; Chen et al., 2023). Gebauer and Fleisch (2007) claim that servitization enables businesses to make five times as much money from services as they do from products, guaranteeing a consistent flow of income regardless of volatility in the economy and boosting competitiveness. Therefore, businesses can obtain a competitive edge by providing long-term, comprehensive, and high-quality services that are focused on products or customers ( Chen et al., 2023). Furthermore, servitization in the manufacturing sector has a big effect on business performance and global competitiveness. Leading manufacturing companies that implement servitization strategies usually hold onto essential components like production, design, and R&D, which allows them to react quickly to needs from around the world ( Huang et al., 2024).

**5.4.2 Firm performance**

According to Miao et al. (2023), servitization plays a mediating role in industrial digitalization’s enhancement of firm performance. By harnessing the advantages of servitization, digitalization eventually boosts business performance. According to Martín-Peña et al. (2020), servitization improves customer interactions, lowers costs, increases efficiency, and provides integrated packages by augmenting digitalization. In a similar vein, Bortoluzzi et al. (2022) found that servitization and firm success were positively correlated. But regardless of a company’s level of technical maturity, Rakic et al. (2022) discovered that only Big Data-based digital solutions had a favorable and meaningful impact on business performance. However, in order to fully comprehend the effects of digital servitization, it is important to recognize a number of services that have a detrimental and significant influence on the performance of the firm. Examples of these services include software development, digital solutions, and PRS (Product-Related Services) in low-tech companies.

**5.4.3 Financial performance**

A company’s ability to create income, turn a profit, and increase its market value is referred to as its financial performance ( Westerman et al., 2014). A competitive advantage and maybe better financial performance can result from strategic adjustments made to a business model to make it more customer-centric (by servitization) and data-driven (via digitalization) ( Abou-Foul et al., 2021). According to Abou-Foul et al. (2021), there is a possibility for digitalization and data-driven solutions to improve financial performance and servitization. This might lead to the development of more effective market offerings. To realize the benefits of digitalization and attain positive performance effects, manufacturing organizations may need to invest in portfolios of sophisticated services, as digitalization alone may not be sufficient to produce favorable financial performance effects ( Kohtamäki et al., 2020). To maximize the benefits of digitalization and improve the financial performance of manufacturing firms, servitization is a prerequisite ( Kohtamäki et al., 2020).

**5.4.4 Firm profitability**

By offering unique product-service combinations, sustainable business models and servitization-related competencies have the potential to increase a company’s competitive edge and profitability ( Kohtamäki et al., 2024). According to Kohtamäki et al. (2024), a focus on sustainability has a direct negative effect on a company’s profitability. However, servitization plays a positive moderating influence, highlighting its importance in producing profits from this concentration on sustainability. Additionally, Vendrell-Herrero et al. (2023) contend that dual innovation enterprises have higher profitability when they use digital service innovation.

**5.4.5 Sustainable performance**

In today’s rapidly evolving business environment, digital servitization emerges as a pivotal strategy for firms aiming to enhance their sustainable performance ( Paiola et al., 2021). By offering value-added services aligned with customer needs, organizations can align their objectives, leading to heightened customer satisfaction and overall performance. Through servitization, firms can cultivate enduring customer relationships, fostering a sustainable revenue stream crucial for sustained performance. Moreover, servitization propels product enhancement efforts, as companies continuously strive to improve their offerings to meet evolving customer demands ( Behl et al., 2024). Achieving sustainable performance necessitates companies to adapt to market dynamics and prioritize delivering solutions that address customer needs. Behl et al. (2024) argue that digital servitization positively influences a firm’s sustainable performance.

Pham and Vu (2022) define sustainable-oriented organizational performance as an organization’s capacity to efficiently and effectively utilize available resources to understand the interconnectedness of ecological, social, and economic dimensions of sustainability. They illustrate how digital servitization is becoming increasingly vital to organizational success in a sustainable manner by outlining its impact on sustainable-oriented organizational performance. Undoubtedly, a focused approach on these opportunities would enhance operational efficiency ( Coreynen et al., 2017).

## 6. Implications

### 6.1 Practical implications

For business practitioners, the findings underscore the critical importance of adopting digital technologies and transitioning to service-centric business models to enhance operational efficiency and customer value. Companies should invest in IoT, big data, and cloud computing to create intelligent products and services while developing capabilities related to digitization, servitization, and innovation. This strategic transformation can improve customer experience through better monitoring, control, and optimization of services, leading to higher customer satisfaction and loyalty. Additionally, integrating digital and service-oriented strategies can help businesses achieve sustainable competitive advantages, enhancing their long-term profitability and market position.

For policymakers, the study highlights the need to create and implement regulations that support the adoption of digital servitization. Developing legal frameworks that ensure transparency, efficiency, and investor confidence is crucial. Policymakers should provide incentives for businesses to invest in digital technologies and service-oriented models, such as tax breaks, grants, and other financial support mechanisms. Investing in necessary digital infrastructure, improving internet connectivity, data security, and digital literacy, is also essential. Encouraging collaboration between the public and private sectors can foster innovation and effective implementation of digital servitization strategies by facilitating partnerships and networks that promote knowledge sharing and resource pooling.

### 6.2 Theoretical implications

The study contributes to the expansion of knowledge by highlighting the need for broader research on digital servitization beyond the manufacturing sector. Future studies should explore its application in various industries to provide a comprehensive understanding of its impacts and potential. An interdisciplinary approach, incorporating insights from business management, engineering, and information technology, can enhance the theoretical framework of digital servitization. This can lead to a more holistic understanding of the concept.

Methodologically, the study suggests the inclusion of qualitative and mixed-methods research to capture the experiential and contextual nuances of digital servitization, providing richer, more detailed insights that quantitative methods alone might miss. Longitudinal studies are also recommended to understand the long-term effects and evolution of digital servitization strategies, identifying trends, challenges, and best practices over time. Conceptually, the identification of key determinant and response variables contributes to the theoretical development of digital servitization. Future research should build on these findings to refine and expand the theoretical constructs associated with digital servitization. Exploring the causal relationships between digital servitization and various performance metrics can deepen theoretical insights, strengthening the theoretical foundation of the field.

## 7. Conclusions

In conclusion, adhering to the established inclusion and exclusion criteria, a total of 26 studies were selected and identified. The primary analysis revealed that research related to digital servitization identified five determinant variables: digitization, servitization, manufacturing servitization, process innovation, and product innovation, as well as five response variables: firm competitiveness, firm performance, financial performance, firm profitability, and sustainable performance.

Compared to other studies, the results of this research offer unique perspectives through in-depth comparative analysis and better classification, leading to more detailed and specific understandings of achieving legal harmonization in the context of municipal bond issuance. These findings contribute to the existing body of knowledge by highlighting the importance of legal harmonization in enhancing the effectiveness and transparency of regional development financing, thereby supporting sustainable development through the adoption of proven legal frameworks.

In summary, these findings underscore the significance of comprehending the role of digital servitization in today’s business landscape. The results of this analysis provide valuable insights for business practitioners, policymakers, and researchers interested in developing effective business strategies in the face of digitization and servitization changes. Thus, this research makes a significant contribution to expanding our understanding of digital servitization and its impact on various aspects of company performance.

The limitations of this study lie in its exclusive focus on quantitative methods, leading to the exclusion of qualitative and mixed-methods research. By limiting the scope to quantitative approaches, the review may overlook the contextual nuances and experiential dimensions inherent in the phenomenon of digital servitization. Qualitative data, with its emphasis on in-depth exploration and understanding of individual experiences, organizational contexts, and socio-cultural factors, could offer valuable insights that quantitative analysis alone might miss. Therefore, the absence of qualitative and mixed-methods research in this review may result in a less comprehensive understanding of digital servitization and its implications.

Furthermore, future research could further explore the causal relationships between these variables and consider different industry and geographical contexts. In conclusion, digital servitization is a phenomenon that plays a crucial role in the modern business era, and a better understanding of the influencing factors and their impacts will continue to be an interesting and relevant research area.

## Data Availability

No data associated with this article. Figshare: Biblio Information in Systematic Literature Review in Digital Servitization, https://doi.org/10.6084/m9.figshare.25833901.v1 ( Ginting, 2024c). This project contains the following data:
• Biblio Information SLR Digital Servitization.csv. Figshare: PRISMA 2020_Checklist Hendri Ginting SLR, https://doi.org/10.6084/m9.figshare.25894162.v1 ( Ginting, 2024b). This project contains the following data:
• PRISMA 2020_Checklist Hendri Ginting SLR Data are available under the terms of the Creative Commons “No Rights Reserved” license (CC0) PRISMA checklist and flowchart for “Exploring the Landscape of Digital Servitization: A Systematic Review”. https://doi.org/10.6084/m9.figshare.25894162.v1.

## References

[ref1] Abou-Foul M Ruiz-Alba JL Soares A : The impact of digitalization and servitization on the financial performance of a firm: an empirical analysis. *Prod. Plan. Control.* 2021;32(12):975–989. 10.1080/09537287.2020.1780508

[ref2] Agarwal GK Simonsson J Magnusson M : Value-capture in digital servitization. *J. Manuf. Technol. Manag.* 2022;33(5):986–1004. 10.1108/JMTM-05-2021-0168

[ref3] Barney J : Firm resources and sustained competitive advantage. *J. Manag.* 1991;17(1):99–120. 10.1177/014920639101700108

[ref4] Behl A Sampat B Gaur J : Can gamification help green supply chain management firms achieve sustainable results in servitized ecosystem? An empirical investigation. *Technovation.* 2024;129:102915. 10.1016/j.technovation.2023.102915

[ref5] Bortoluzzi G Chiarvesio M Romanello R : Servitisation and performance in the business-to-business context: the moderating role of Industry 4.0 technologies. *J. Manuf. Technol. Manag.* 2022;33(9):108–128. 10.1108/JMTM-08-2021-0317

[ref6] Bromiley P Papenhausen C : Assumptions of Rationality and Equilibrium in Strategy Research: The Limits of Traditional Economic Analysis. *Strateg. Organ.* 2003;1(4):413–437. 10.1177/14761270030014003

[ref7] Bustinza OF Gomes E Vendrell-Herrero F : An organizational change framework for digital servitization: Evidence from the Veneto region. *Strateg. Chang.* 2018;27(2):111–119. 10.1002/jsc.2186

[ref8] Chen L Dai Y Ren F : Data-driven digital capabilities enable servitization strategy——From service supporting the product to service supporting the client. *Technol. Forecast. Soc. Chang.* 2023;197:122901. 10.1016/j.techfore.2023.122901

[ref9] Chen S Zhang H : Does digital finance promote manufacturing servitization: Micro evidence from China. *Int. Rev. Econ. Financ.* 2021;76:856–869. 10.1016/j.iref.2021.07.018

[ref10] Chen Y Visnjic I Parida V : On the road to digital servitization – The (dis) continuous interplay between business model and digital technology. *Int. J. Oper. Prod. Manag.* 2021;41(5):694–722. 10.1108/IJOPM-08-2020-0544

[ref11] Chirumalla K Leoni L Oghazi P : Moving from servitization to digital servitization: Identifying the required dynamic capabilities and related microfoundations to facilitate the transition. *J. Bus. Res.* 2023;158(January):113668. 10.1016/j.jbusres.2023.113668

[ref12] Coreynen W Matthyssens P Van Bockhaven W : Boosting servitization through digitization: Pathways and dynamic resource configurations for manufacturers. *Ind. Mark. Manag.* 2017;60:42–53.

[ref13] Coreynen W Matthyssens P Vanderstraeten J : Unravelling the internal and external drivers of digital servitization: A dynamic capabilities and contingency perspective on firm strategy. *Ind. Mark. Manag.* 2020;89(December 2018):265–277. 10.1016/j.indmarman.2020.02.014

[ref14] Cruzes DS Dyba T : Recommended steps for thematic synthesis in software engineering. *2011 International Symposium on Empirical Software Engineering and Measurement.* IEEE;2011, September; pp.275–284.

[ref15] Farisyi S Musadieq MA Utami HN : A systematic literature review: Determinants of sustainability reporting in developing countries. *Sustainability.* 2022;14(16):10222. 10.3390/su141610222

[ref16] Frank AG Mendes GHS Ayala NF : Servitization and Industry 4.0 convergence in the digital transformation of product firms: A business model innovation perspective. *Technol. Forecast. Soc. Chang.* 2019;141(July 2018):341–351. 10.1016/j.techfore.2019.01.014

[ref17] Gao J Zhang W Guan T : Influence of digital transformation on the servitization level of manufacturing SMEs from static and dynamic perspectives. *Int. J. Inf. Manag.* 2023;73:102645. 10.1016/j.ijinfomgt.2023.102645

[ref18] Garcia R Calantone R : A critical look at technological innovation typology and innovativeness terminology: a literature review. *Journal of Product Innovation Management: An International Publication of the Product Development & Management Association.* 2002;19(2):110–132. 10.1111/1540-5885.1920110

[ref19] Gebauer H Fleisch E : An investigation of the relationship between behavioral processes, motivation, investments in the service business and service revenue. *Ind. Mark. Manag.* 2007;36(3):337–348. 10.1016/j.indmarman.2005.09.005

[ref20] Gebauer H Gustafsson A Witell L : Service transition in manufacturing companies: a capabilities perspective. *J. Bus. Strateg.* 2020;41(3):51–58.

[ref21] Ginting H : Flowchart illustrating the process of literature selection reviewed using PRISMA. figshare. *Media.* 2024a. 10.6084/m9.figshare.25894591.v1

[ref22] Ginting H : PRISMA 2020_Checklist Hendri Ginting SLR.Dataset. *figshare.* 2024b. 10.6084/m9.figshare.25894162.v1

[ref23] Ginting H : Biblio Information SLR Digital Servitization.csv.Dataset. *figshare.* 2024c. 10.6084/m9.figshare.25833901.v1

[ref24] Hacioglu U : *Handbook of research on strategic fit and design in business ecosystems.* IGI Global;2019.

[ref25] Hilbolling S Deken F Berends H : Process-based temporal coordination in multiparty collaboration for societal challenges. *Strateg. Organ.* 2022;20(1):135–163. 10.1177/1476127021992705

[ref26] Huang G Ma L Xietian Z : Servitization of manufacturing and China’s power status upgrading of global value network. *Struct. Chang. Econ. Dyn.* 2024;68:313–328. 10.1016/j.strueco.2023.11.005

[ref27] Huikkola T Kohtamäki M : Solution providers’ strategic capabilities. In. *J. Bus. Ind. Mark.* 2017;32(5):752–770. 10.1108/JBIM-11-2015-0213

[ref28] Jiang S Hu X Li S : Effect of manufacturing service transformation management on technological innovation. *J. Innov. Knowl.* 2023;8(4):100404. 10.1016/j.jik.2023.100404

[ref29] Kohtamäki M Bhandari KR Rabetino R : Sustainable servitization in product manufacturing companies: The relationship between firm’s sustainability emphasis and profitability and the moderating role of servitization. *Technovation.* 2024;129:102907. 10.1016/j.technovation.2023.102907

[ref30] Kohtamäki M Parida V Oghazi P : Digital servitization business models in ecosystems: A theory of the firm. *J. Bus. Res.* 2019;104(June):380–392. 10.1016/j.jbusres.2019.06.027

[ref31] Kohtamäki M Parida V Patel PC : The relationship between digitalization and servitization: The role of servitization in capturing the financial potential of digitalization. *Technol. Forecast. Soc. Chang.* 2020;151(July 2019):119804. 10.1016/j.techfore.2019.119804

[ref32] Kowalkowski C Gebauer H Kamp B : Servitization and deservitization: Overview, concepts, and definitions. *Ind. Mark. Manag.* 2017;60:4–10. 10.1016/j.indmarman.2016.12.007

[ref33] Kraja YB : The impact of tangible and intangible assets on the SMEs’ success: the Albanian case. *Entrepreneurship in Post-Communist Countries: New Drivers Towards a Market Economy.* 2018;135–145. 10.1007/978-3-319-75907-4_9

[ref34] Kumar M Raut RD Mangla SK : Moderating ESG compliance between industry 4.0 and green practices with green servitization: Examining its impact on green supply chain performance. *Technovation.* 2024;129:102898. 10.1016/j.technovation.2023.102898

[ref35] Lalic B Marjanovic U Rakic S : Big data analysis as a digital service: evidence form manufacturing firms. *Proceedings of 5th International Conference on the Industry 4.0 Model for Advanced Manufacturing: AMP 2020.* 2020; pp.263–269. 10.1007/978-3-030-46212-3_19

[ref36] Leiblein MJ : The choice of organizational governance form and performance: Predictions from transaction cost, resource-based, and real options theories. *J. Manag.* 2003;29(6):937–961. 10.1016/S0149-2063(03)00085-0

[ref37] Lerch C Gotsch M : Digitalized product-service systems in manufacturing firms: A case study analysis. *Res. Technol. Manag.* 2015;58(5):45–52. 10.5437/08956308X5805357

[ref38] Liberati A Altman DG Tetzlaff J : The PRISMA statement for reporting systematic reviews and meta-analyses of studies that evaluate health care interventions: explanation and elaboration. *Ann. Intern. Med.* 2009;151(4):W-65. 19622512 10.7326/0003-4819-151-4-200908180-00136

[ref39] Martín-Peña ML Díaz-Garrido E Sánchez-López JM : The digitalization and servitization of manufacturing: A review on digital business models. *Strateg. Chang.* 2018;27(2):91–99. 10.1002/jsc.2184

[ref40] Martín-Peña ML Sánchez-López JM Díaz-Garrido E : Servitization and digitalization in manufacturing: the influence on firm performance. *J. Bus. Ind. Mark.* 2020;35(3):564–574. 10.1108/JBIM-12-2018-0400

[ref41] Miao Y Shi Y Jing H : Effect of servitization on performance in manufacturing firms: A mediating effect model of digitalisation moderated by ESG performance. *Heliyon.* 2023;9(10):e20831. 10.1016/j.heliyon.2023.e20831 37886777 PMC10597834

[ref42] Mishra V Bisoyi B Das B : Supply Chain Transformation Through Digital Servitization in Manufacturing Sector. *Recent Advances in Thermofluids and Manufacturing Engineering: Select Proceedings of ICTMS 2022.* Springer;2022; pp.571–579.

[ref43] Moher D Shamseer L Clarke M : Preferred reporting items for systematic review and meta-analysis protocols (PRISMA-P) 2015 statement. *Syst. Rev.* 2015;4:1–9. 10.1186/2046-4053-4-1 25554246 PMC4320440

[ref44] Neely A : Exploring the financial consequences of the servitization of manufacturing. *Oper. Manag. Res.* 2008;1(2):103–118. 10.1007/s12063-009-0015-5

[ref45] Opazo-Basáez M Vendrell-Herrero F Bustinza OF : Digital service innovation: a paradigm shift in technological innovation. *J. Serv. Manag.* 2022;33(1):97–120. 10.1108/JOSM-11-2020-0427

[ref46] Paiola M Schiavone F Grandinetti R : Digital servitization and sustainability through networking: Some evidences from IoT-based business models. *J. Bus. Res.* 2021;132:507–516. 10.1016/j.jbusres.2021.04.047

[ref47] Parate V Hiremath BV Tailor RK : Digital servitization: An effective tool of attracting customers in modern business world. *J. Manag. Res. Anal.* 2022;9(4):207–209. 10.18231/j.jmra.2022.041

[ref48] Paschou T Rapaccini M Adrodegari F : Digital servitization in manufacturing: A systematic literature review and research agenda. *Ind. Mark. Manag.* 2020;89:278–292. 10.1016/j.indmarman.2020.02.012

[ref49] Penrose ET : *The Theory of the Growth of the Firm.* Oxford: Oxford University Press;1959.

[ref50] Pham HQ Vu PK : Unravelling the Potential of Digital Servitization in Sustainability-Oriented Organizational Performance—Does Digital Leadership Make It Different? *Economies.* 2022;10(8):185. 10.3390/economies10080185

[ref51] Porter ME : Competitive strategy. *Meas. Bus. Excell.* 1997;1(2):12–17. 10.1108/eb025476

[ref52] Raddats C Burton J Zolkiewski J : Servitization capabilities for advanced services: a multi-actor perspective. *Second Spring Servitization Conference.* 2014; pp.126–132.

[ref53] Rakic S Pero M Sianesi A : Digital servitization and firm performance: Technology intensity approach. *Inzinerine Ekonomika.* 2022;33(4):398–413.

[ref54] Rymaszewska A Helo P Gunasekaran A : IoT powered servitization of manufacturing – an exploratory case study. *Int. J. Prod. Econ.* 2017;192:92–105. 10.1016/j.ijpe.2017.02.016

[ref55] Schumpeter JA Swedberg R : *The theory of economic development.* Routledge;2021. 10.4324/9781003146766

[ref56] Sklyar A Kowalkowski C Tronvoll B : Organizing for digital servitization: A service ecosystem perspective. *J. Bus. Res.* 2019;104(October 2017):450–460. 10.1016/j.jbusres.2019.02.012

[ref57] Tronvoll B Sklyar A Sörhammar D : Transformational shifts through digital servitization. *Ind. Mark. Manag.* 2020;89(February):293–305. 10.1016/j.indmarman.2020.02.005

[ref58] Upadhayay NB Rocchetta S Gupta S : Blazing the trail: The role of digital and green servitization on technological innovation. *Technovation.* 2024;130(December):102922. 10.1016/j.technovation.2023.102922

[ref59] Valtakoski A : Explaining servitization failure and deservitization: A knowledge-based perspective. *Ind. Mark. Manag.* 2017;60:138–150. 10.1016/j.indmarman.2016.04.009

[ref60] Vargo SL Lusch RF : Service-dominant logic 2025. *Int. J. Res. Mark.* 2017;34(1):46–67. 10.1016/j.ijresmar.2016.11.001

[ref61] Vendrell-Herrero F Bustinza OF Opazo-Basaez M : Treble innovation firms: Antecedents, outcomes, and enhancing factors. *Int. J. Prod. Econ.* 2023;255:108682. 10.1016/j.ijpe.2022.108682

[ref62] Vendrell-Herrero F Bustinza OF Parry G : Servitization, digitization and supply chain interdependency. *Ind. Mark. Manag.* 2017;60:69–81. 10.1016/j.indmarman.2016.06.013

[ref63] Visnjic I Neely A Jovanovic M : The path to outcome delivery: Interplay of service market strategy and open business models. *Technovation.* 2018;72-73(February):46–59. 10.1016/j.technovation.2018.02.003

[ref64] Wang J Wang W Wu H : Exploring the effects of manufacturing servitization on enterprise energy conservation and emissions reduction moderated by digital transformation. *Energy Econ.* 2023;122:106706. 10.1016/j.eneco.2023.106706

[ref65] Wen H Wen C Lee C-C : Impact of digitalization and environmental regulation on total factor productivity. *Inf. Econ. Policy.* 2022;61:101007. 10.1016/j.infoecopol.2022.101007

[ref66] Wernerfelt B : On the role of the RBV in marketing. *J. Acad. Mark. Sci.* 2014;42(1):22–23. 10.1007/s11747-013-0335-8

[ref67] Westerman G Bonnet D McAfee A : *Leading digital: Turning technology into business transformation.* Harvard Business Press;2014.

[ref68] Zhao S Peng D Wen H : Does the digital economy promote upgrading the industrial structure of Chinese cities? *Sustainability.* 2022;14(16):10235. 10.3390/su141610235

[ref69] Zhou D Wu Q Lee S : Unpacking the mechanism linking digital servitization and manufacturing firm performance: the role of the service networks and slack resources. *J. Bus. Ind. Mark.* 2023.

