# Supplementary material for: Exploring the landscape of digital servitization: A systematic review
Source: F1000Res. 2024 Sep 25;13:813. Originally published 2024 Jul 18. [Version 2] doi: 10.12688/f1000research.150946.2 (PMC11628934; doi:10.12688/f1000research.150946.2)
Supplement: Peer review history

# Reviewer report: Reviewer response for version 2

**DOI:** 10.5256/f1000research.172105.r338312

**Published:** 2024-12-09

**Authors:** Martin Wynn

**Affiliations:** 1 University of Gloucestershire, Cheltenham, UK

This is a very interesting and topical article. It is generally well constructed and well written, and is fine as far as it goes. Often with systematic literature reviews, there are some aspects of potential discussion missing because of the selection of search strings. Here, there is a body of literature on the differences and distinctions between digialization and digital transformation that I think would be very relevant and worth discussion. For example, a number of articles have suggested that digitalisation becomes digital transformation when digital technologies are used either within/as part of the company's products OR become embedded in the companies services. This concept of digital servitization meets the second criteria, and in my book thus is one form of digital transformation. The other could be called product digitalisation. Some articles that might be worth considering that address this include:

 Abdallah-Ou-Moussa, Soukaina, Wynn, Martin G , Kharbouch, Omar and Rouaine, Zakaria (2024) Digitalization and Corporate Social Responsibility: A Case Study of the Moroccan Auto Insurance Sector. Administrative Sciences, 14 (282). Ref 1

 Wynn, Martin G  and Felser, Kerstin  (2023) Digitalisation and Change in the Management of IT. Computers, 12 (12). Art 251. Ref 2

 Wynn, Martin G  and Lam, Carmen (2023) Digitalisation and IT strategy in the hospitality industry. Systems, 11 (10). Art 501.Ref 3 

 The relevance of digital servitization in manufacturing and other industries to the debate about what constitutes digital transformation could be included in the discussion or conclusion sections.

 Overall, this is a very useful article and I think is worthy of indexing.

Are the rationale for, and objectives of, the Systematic Review clearly stated?

Yes

Is the statistical analysis and its interpretation appropriate?

Yes

If this is a Living Systematic Review, is the ‘living’ method appropriate and is the search schedule clearly defined and justified? (‘Living Systematic Review’ or a variation of this term should be included in the title.)

Yes

Are sufficient details of the methods and analysis provided to allow replication by others?

Yes

Are the conclusions drawn adequately supported by the results presented in the review?

Yes

Reviewer Expertise:

Digitalisation; IT strategy; sustainability

I confirm that I have read this submission and believe that I have an appropriate level of expertise to confirm that it is of an acceptable scientific standard.

## References

[1] : Digitalization and Corporate Social Responsibility: A Case Study of the Moroccan Auto Insurance Sector. *Administrative Sciences* .2024;14(11) : 10.3390/admsci14110282 10.3390/admsci14110282

[2] : The Role of Digitalisation in Shaping a Country's Image. *European Conference on Cyber Warfare and Security* .2024;23(1) : 10.34190/eccws.23.1.2404 1-9 10.34190/eccws.23.1.2404

[3] : Digitalisation and IT Strategy in the Hospitality Industry. *Systems* .2023;11(10) : 10.3390/systems11100501 10.3390/systems11100501

# Reviewer report: Reviewer response for version 2

**DOI:** 10.5256/f1000research.172105.r326913

**Published:** 2024-10-25

**Authors:** João Reis

**Affiliations:** 1 Universidade Lusófona, Lisbon, Portugal

The authors responded appropriately to the reviewer's suggestions.

Are the rationale for, and objectives of, the Systematic Review clearly stated?

Partly

Is the statistical analysis and its interpretation appropriate?

Partly

If this is a Living Systematic Review, is the ‘living’ method appropriate and is the search schedule clearly defined and justified? (‘Living Systematic Review’ or a variation of this term should be included in the title.)

Not applicable

Are sufficient details of the methods and analysis provided to allow replication by others?

Partly

Are the conclusions drawn adequately supported by the results presented in the review?

Partly

Reviewer Expertise:

Industrial Engineering; Digital Transformation

I confirm that I have read this submission and believe that I have an appropriate level of expertise to confirm that it is of an acceptable scientific standard.

# Reviewer report: Reviewer response for version 1

**DOI:** 10.5256/f1000research.165562.r307042

**Published:** 2024-08-07

**Authors:** João Reis

**Affiliations:** 1 Universidade Lusófona, Lisbon, Portugal

I was kind of excited to read the article titled “Exploring the Landscape of Digital Servitization: A Systematic Review.”

 This article is particularly important due to its relevance and the increasing interest it has gained in the academic community. It holds the potential to provide a comprehensive view of digital servitization within the context of fundamental sciences. However, several improvements are needed to improve its quality.

 Firstly, it is crucial to update the references during the revision process. Since the submission of the manuscript, several relevant articles on digital servitization have been published, such as:

  

1. Soellner, S., et al. (2024). [Ref-1].
2. Leocádio, D., et al., (2024). [Ref-2].

It is understandable that the references are not up-to-date as of 2024 due to the research date and the often lengthy review process. However, updating these references before indexing can enhance the article's relevance.

 Additionally, the Preferred Reporting Items for Systematic Reviews and Meta-Analyses (PRISMA) currently includes 3 phases, not 4 as indicated in Figure 1. This needs to be corrected, and I recommend consulting the official website: https://www.prisma-statement.org

 The figures in the article require a more professional layout. For example, Figure 2 is barely visible and should be improved.

 The discussion section would benefit from continuous text and fewer bullet points. Excessive bullet points disrupt the logical flow, making the content seem fragmented. A coherent chain of ideas, transitioning from the most general to the most specific, is more effective (I believe).

 The article's structure, which currently includes 7 headings, feels overly fragmented. Adopting the IMRaD (Introduction, Method, Results, and Discussion) format, which is well-established in the academic community, can provide a clearer and more cohesive structure to your article.

 Furthermore, the article needs extensive English proofreading with a focus on scientific writing. While I agree that using AI tools for proofreading is acceptable, human refinement is still essential in this context to ensure the writing meets scientific standards.

 Although my recommendations might seem broad at this stage, they are intended to help the authors develop a more robust article. If the authors agree to revise their article, I am willing to provide more detailed feedback before final approval.

 The authors are not obliged to cite the articles above, as long as they cite current articles that are strictly related to the topic

 Good luck and have good work!

Are the rationale for, and objectives of, the Systematic Review clearly stated?

Partly

Is the statistical analysis and its interpretation appropriate?

Partly

If this is a Living Systematic Review, is the ‘living’ method appropriate and is the search schedule clearly defined and justified? (‘Living Systematic Review’ or a variation of this term should be included in the title.)

Not applicable

Are sufficient details of the methods and analysis provided to allow replication by others?

Partly

Are the conclusions drawn adequately supported by the results presented in the review?

Partly

Reviewer Expertise:

Industrial Engineering; Digital Transformation

I confirm that I have read this submission and believe that I have an appropriate level of expertise to confirm that it is of an acceptable scientific standard, however I have significant reservations, as outlined above.

## References

[1] : Industrial service innovation: Exploring the transformation process to digital servitization in industrial goods companies. *Industrial Marketing Management* .2024;117: 10.1016/j.indmarman.2024.01.009 288-303 10.1016/j.indmarman.2024.01.009

[2] : Servitization in Digital Age: A Systematic Literature Review. *Procedia Computer Science* .2024;232: 10.1016/j.procs.2024.02.071 2531-2539 10.1016/j.procs.2024.02.071

# Response

**Published:** 2024-09-18

**Authors:** Hendri Ginting

**Affiliations:** Universitas Brawijaya, Malang, East Java, Indonesia

1. the reference has been updated using the most recent references:
  "The concept of servitization spans a range between pure providers of tangible goods and pure providers of services. Within this spectrum, manufacturing companies are moving in the direction of service providers … (Soellner, S., et al., 2024)."
  (page 2)
2. the Preferred Reporting Items for Systematic Reviews and Meta-Analyses (PRISMA) currently includes 3 phases, not 4 as indicated in Figure 1. This needs to be corrected, and I recommend consulting the official website: https://www.prisma-statement.org --> Revised on Page 6 and the picture is also attached on separated file
3. The figures in the article require a more professional layout. For example, Figure 2 is barely visible and should be improved. --> revised on Page 7 and the picture is also attached on separated file
4. The discussion section would benefit from continuous text and fewer bullet points. Excessive bullet points disrupt the logical flow, making the content seem fragmented. A coherent chain of ideas, transitioning from the most general to the most specific, is more effective (I believe) --> The discussion section revised into continous text
5. The article’s structure has been simplified into 4 sections adopting the IMRaD (Introduction, Method, Results, and Discussion)
6. This paper has passed the English grammar check using AI tools and also has been checked by human
